# Reproducibility of In-Vivo OCT Measured Three-Dimensional Human Lamina Cribrosa Microarchitecture

**DOI:** 10.1371/journal.pone.0095526

**Published:** 2014-04-18

**Authors:** Bo Wang, Jessica E. Nevins, Zach Nadler, Gadi Wollstein, Hiroshi Ishikawa, Richard A. Bilonick, Larry Kagemann, Ian A. Sigal, Ireneusz Grulkowski, Jonathan J. Liu, Martin Kraus, Chen D. Lu, Joachim Hornegger, James G. Fujimoto, Joel S. Schuman

**Affiliations:** 1 Department of Ophthalmology, University of Pittsburgh Medical Center (UPMC) Eye Center, Eye and Ear Institute, Ophthalmology and Visual Science Research Center, University of Pittsburgh School of Medicine, Pennsylvania, United States of America; 2 Department of Bioengineering, Swanson School of Engineering, University of Pittsburgh, Pittsburgh, Pennsylvania, United States of America; 3 Department of Biostatistics, Graduate School of Public Health, University of Pittsburgh, Pittsburgh, Pennsylvania, United States of America; 4 Department of Electrical Engineering and Computer Science, Massachusetts Institute of Technology (MIT), Cambridge, Massachusetts, United States of America; 5 Department of Computer Science, University of Erlangen-Nuremberg, Nuremberg, Germany; Justus-Liebig-University Giessen, Germany

## Abstract

**Purpose:**

To determine the reproducibility of automated segmentation of the three-dimensional (3D) lamina cribrosa (LC) microarchitecture scanned in-vivo using optical coherence tomography (OCT).

**Methods:**

Thirty-nine eyes (8 healthy, 19 glaucoma suspects and 12 glaucoma) from 49 subjects were scanned twice using swept-source (SS−) OCT in a 3.5×3.5×3.64 mm (400×400×896 pixels) volume centered on the optic nerve head, with the focus readjusted after each scan. The LC was automatically segmented and analyzed for microarchitectural parameters, including pore diameter, pore diameter standard deviation (SD), pore aspect ratio, pore area, beam thickness, beam thickness SD, and beam thickness to pore diameter ratio. Reproducibility of the parameters was assessed by computing the imprecision of the parameters between the scans.

**Results:**

The automated segmentation demonstrated excellent reproducibility. All LC microarchitecture parameters had an imprecision of less or equal to 4.2%. There was little variability in imprecision with respect to diagnostic category, although the method tends to show higher imprecision amongst healthy subjects.

**Conclusion:**

The proposed automated segmentation of the LC demonstrated high reproducibility for 3D LC parameters. This segmentation analysis tool will be useful for in-vivo studies of the LC.

## Introduction

The mechanical theory of glaucoma hypothesized that increased intra-ocular pressure (IOP) resulted in deformation of the lamina cribrosa (LC), a matrix of connective tissue within the optic nerve through which all the axons of the retina pass. Previous histology studies demonstrated that changes in the structure of the LC were associated with glaucoma[1]–[3] and finite modeling identified the LC as a potential location of mechanical weakness in the eye. [4], [5].

Recently, there had been an increased interest in in-vivo imaging of the LC using both OCT and scanning laser ophthalmoscopy (SLO).[6]–[9] Due to its higher axial resolution compared to SLO, OCT offer considerable advantages for 3D evaluation of the LC, being able to provide dense optical sampling of the tissue. Unlike histology studies, thorough in-vivo 3D LC studies do not suffer from distortions in tissue preparation, tissue degradation or loss of pressure after death. In-vivo analysis also permits longitudinal assessment of the microarchitecture changes with time and disease. Several current in-vivo OCT studies of the LC had been focused on surface defects, [10] total LC thickness [9] and shape of the anterior LC. [11] However, little work had been done to analyze the LC 3D microarchitecture in-vivo.

One major limitation in studying the LC 3D microarchitecture is the complexity of its structure. Manual segmentation of individual LC pores and beams is time consuming and infeasible for larger studies. We have previously demonstrated an automated method of segmenting and analyzing the 3D microarchitecture features of the LC from OCT imaging, which could significantly reduce the time to analyze the LC. [12] In this study, we assessed the reproducibility of the automated segmentation and analysis of in-vivo LC 3D microarchitecture scanned using OCT.

## Methods

### Patient Population

The study was conducted in accordance with the tenets of the Declaration of Helsinki and the Healthy Insurance Portability and Accountability Act. The institutional review board of the University of Pittsburgh approved this study and all subjects gave written consent before participation.

A total of 39 eyes (8 healthy, 19 glaucoma suspect, 12 glaucoma) from 49 subjects representing the range of healthy and diseased eyes typically seen in glaucoma practice, were enrolled to the study. Eligible subjects had comprehensive ophthalmic examination, intraocular pressure reading, Swedish interactive thresholding algorithm 24-2 visual fields (Humphrey field analyzer; Zeiss, Dublin, CA) and repetitive swept-source (SS−) OCT scanning of the LC. Healthy eyes were characterized as having normal appearance of the optic nerve head (ONH) and retinal nerve fiber layer (RNFL), full visual fields without any previous history of retinal diseases or glaucoma. Glaucomatous eyes were classified based on clinical examination findings characteristics for glaucoma (ONH abnormality – neuroretinal rim thinning, rim notch, or disc hemorrhage; RNFL defect) accompanied with typical glaucomatous visual field loss (reproducible glaucoma hemifield test outside normal limits). Glaucoma suspects were defined as subjects with glaucomatous optic disc appearance, as defined above, in the presence of normal visual field. If both eyes were eligible, they were included in the appropriate category.

### Image Acquisition and Processing with SS-OCT

All subjects underwent 2 SS-OCT scans (3.5 mm×3.5 mm×3.64 mm, 400×400×896 pixels) of the ONH. The two OCT scans were taken within approximately one minute of each other, with the focus and OCT machine readjusted after each scan. The SS-OCT was a prototype device with a scanning speed of 100,000 A-scan/sec, a light source centered at 1050 nm and a 5 µm axial resolution. Between scans, the focus of the OCT scanner was readjusted.

Each SS-OCT scan consisted of two orthogonally oriented (vertical and horizontal) scan volumes. The orthogonal volumes were co-registered to remove motion artifacts. [13] The scans were processed using the FIJI [14] segmentation tool (http://rsbweb.nih.gov/ij/), as previously described. [12] In brief, the segmentation was based upon automated pore and beam segmentation using local thresholds after noise removal. The parameters analyzed include 3D parameters (pore diameter, pore diameter SD, beam thickness, beam thickness SD, beam-pore ratio) and parameters averaged on sequential single pixel thick parallel planes (C-mode slices) (pore area and pore aspect ratio) that were resampled from the scan volume. Pore area represents the average area of every individual pores in the scan volume and pore aspect ratio represents the ratio of the major axis to minor axis of an ellipse fit to each pore. Pore diameter and beam thickness were computed by determining the largest sphere that fit within the structure at any given point. [15] All the pore diameters and beam thicknesses were averaged to generate the mean values.

### Statistical Analysis

Reproducibility was assessed by determining the imprecision SD of repeated measurements using a measurement error model, accounting for the use of both eyes from some of the subjects. [16] The imprecision SD measures the typical size of the random error made by the device when a measurement is made, assuming a bias of 1 since the same method is used to analyze the repeated scans. [17] Relative imprecision was calculated by dividing the imprecision by the measurement’s average. Low imprecision between two scans indicated high reproducibility. Statistical analysis was performed using R Language and Environment for Statistical Computing program (version 2.15.1). [18].

## Results

**Figure 1:**
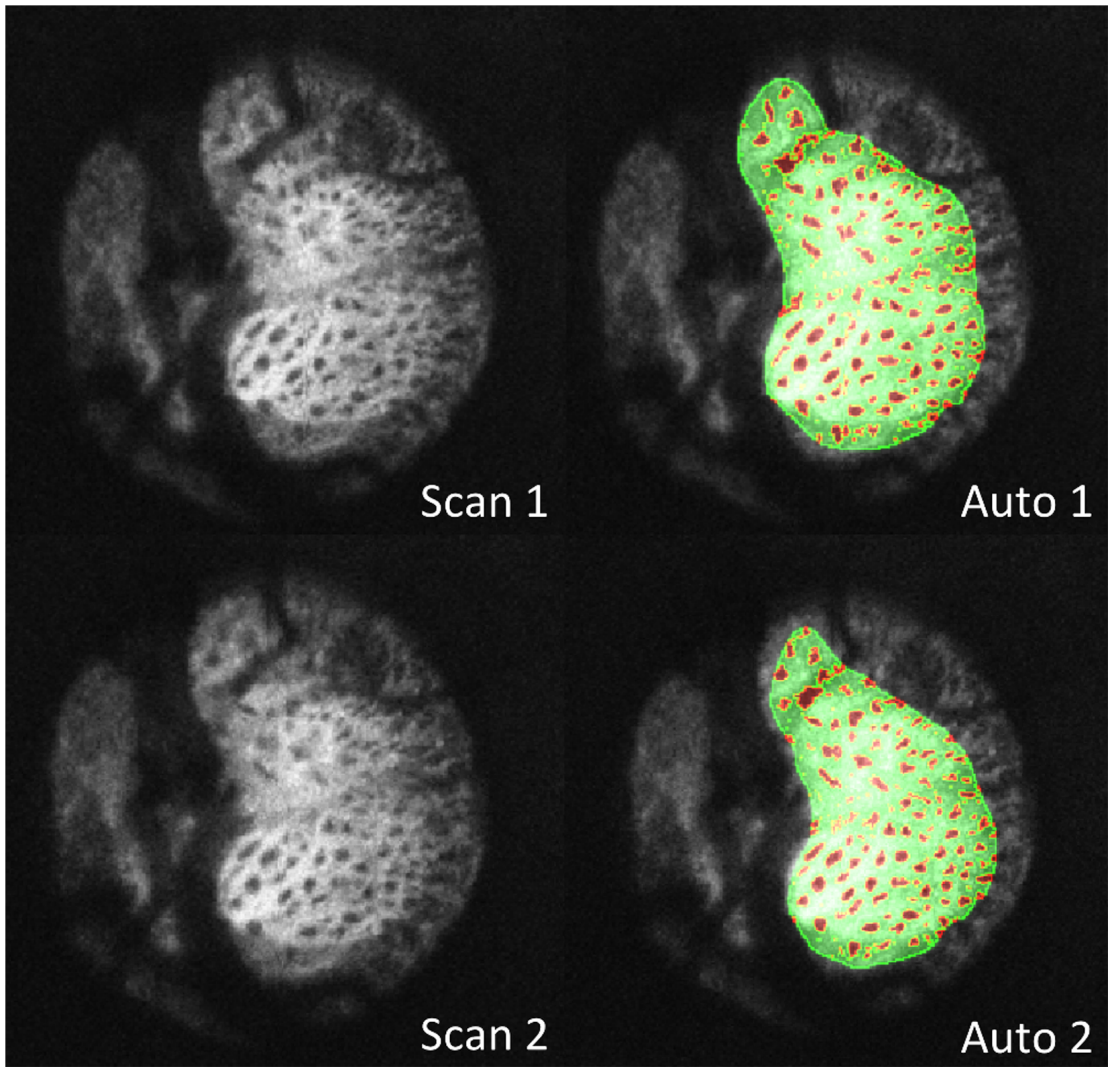
Two consecutive optical coherence tomography scans of the lamina cribrosa of the same eye. Original C-mode (left) and segmentation overlain (right) where beams (green) and pores (red) were marked. The automated demarcation of the pores was highly repeatable between the scans.

**Figure 2:**
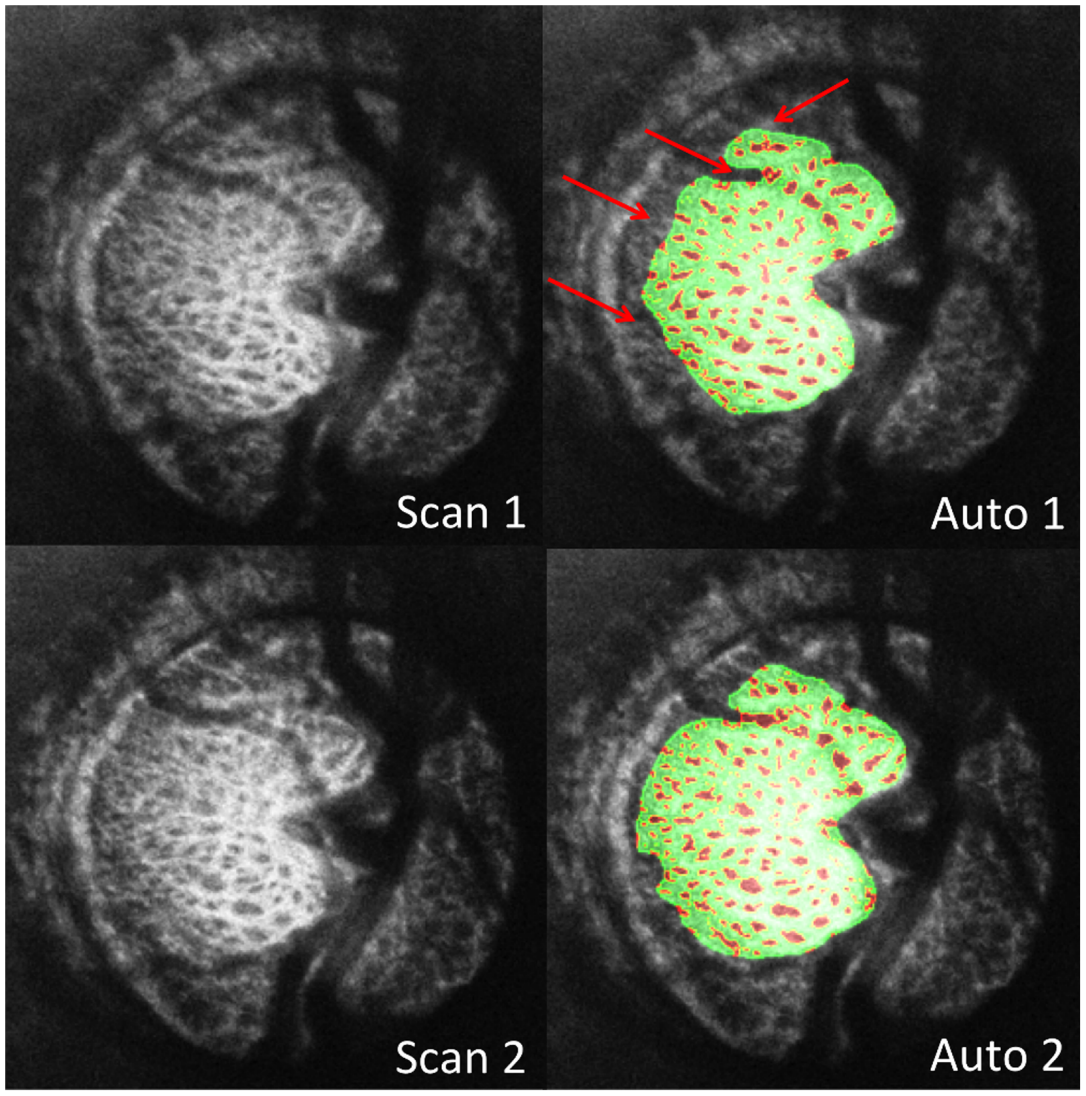
Two consecutive optical coherence tomography scans of the lamina cribrosa of the same eye. Original C-mode (left) and segmentation overlain (right) where beams (green) and pores (red) are marked. Differences in segmentations between the two scans (red arrows) primarily existed due to local disparities in regions analyzed.

**Figure 3:**
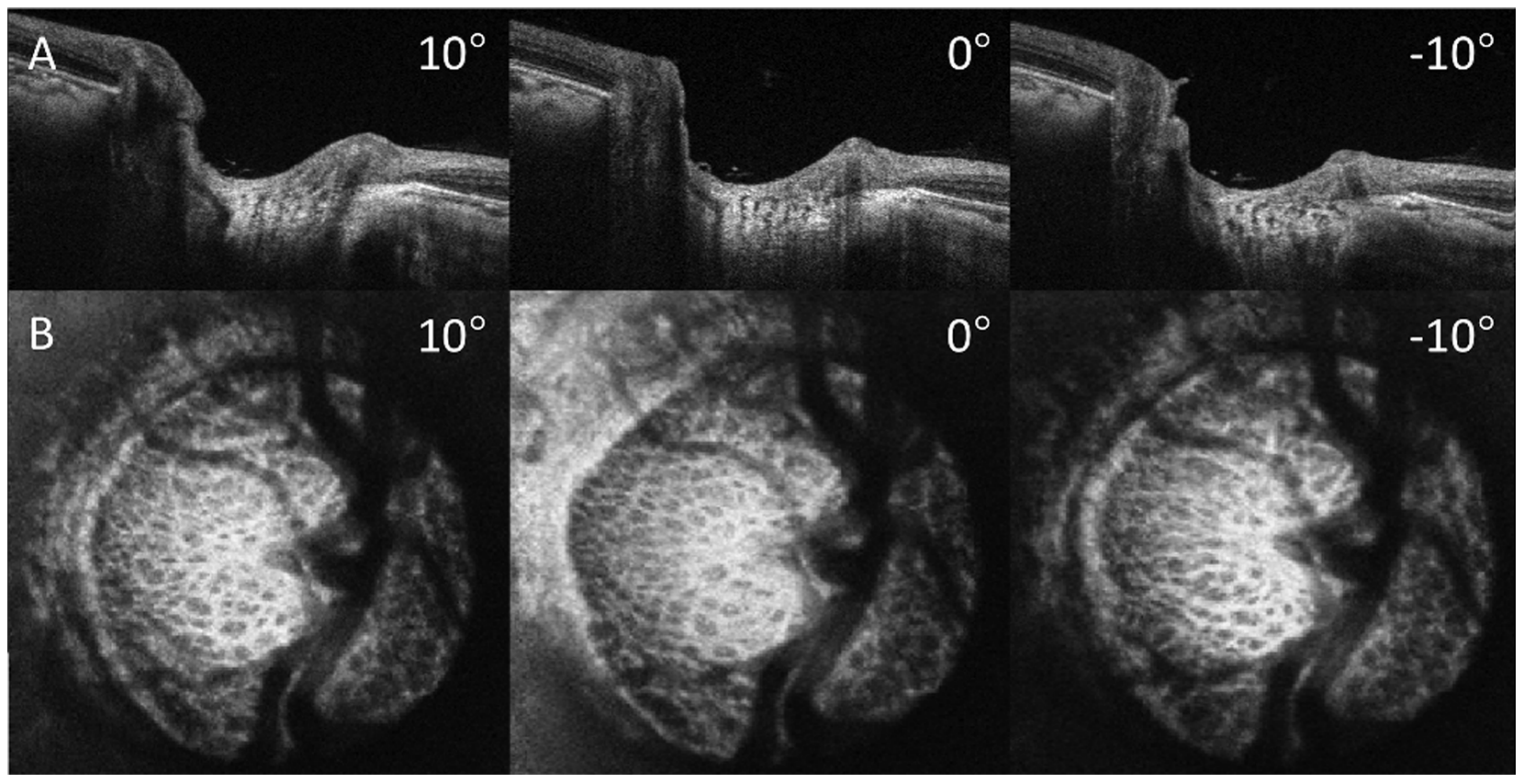
Shifts in the scanning angle alter B-scan (A) and C-mode (B) images and alter the microarchitecture seen on a single frame. OCT volume was rotated ±10° with respect to the slow scanning axis using image processing software (FIJI).

**Figure 4:**
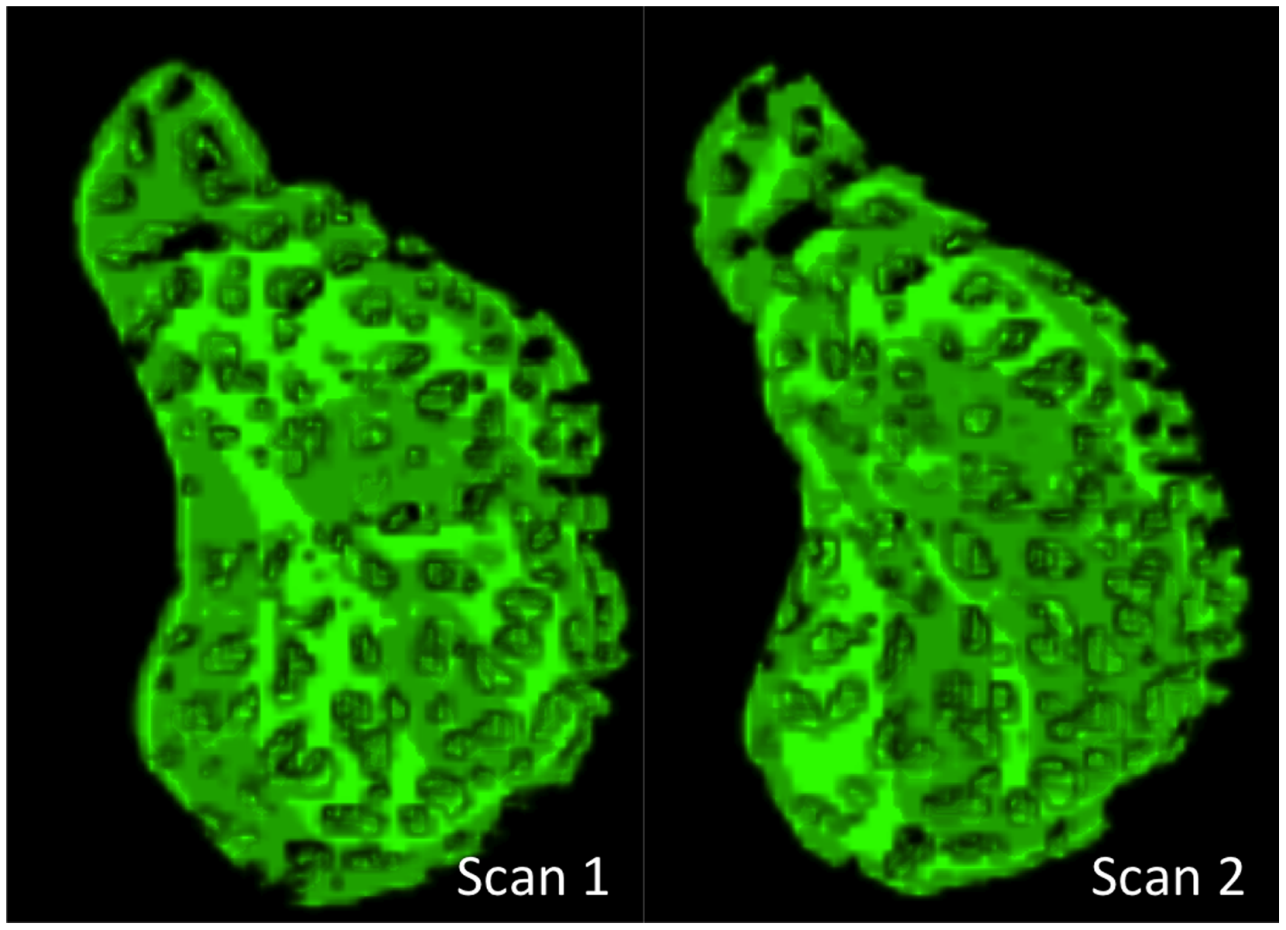
3D view of lamina cribrosa beams demonstrated the reproducibility of the microarchitectural features (same eye as Figure 1).

Average age for all subjects was 57±14 years with an average visual field mean deviation (VF MD) of −2.0±4.9 dB. VF MD based on clinical category was −0.4±0.9 dB for healthy (n  = 8), −0.6±1.3 dB for glaucoma suspects (n  = 19), and −4.5±7.5 dB for glaucoma subjects (n  = 12). Automated segmentation of two different scans of the same eye is shown in Figure 1. Observable differences between automated segmentation of two scans primarily occurred due to differences in region of the LC included in the analysis (Figure 2). These differences could be due to small alterations in scan angle between images creating distortions in the region of LC within a plane (C-mode) and along a cross-section (B-scans) (Figure 3). Despite these edge effects, 3D view of the visible LC appeared highly repeatable (Figure 4). The average number of C-mode slices over which pores were measured was 69±13 slices (range: 38–101). This corresponds to a physical depth of 281±54 µm. The number of pores in the top half of the analyzed LC was 67±29 pores (range: 18–145). The number of pores in the bottom half of the analyzed LC was 54±27 pores (range: 3–114).

The relative imprecision of the parameters is summarized in Table 1. The imprecision of all LC microarchitectural parameters was ≤4.2. Table 2 summarizes the relative imprecision of LC parameters for the different clinical diagnosis groups. There was no significant difference in imprecision between the diagnostic categories with the exception of pore diameter, which was higher in healthy subject compared to glaucoma subjects.

**Table 1:** Average and imprecision for lamina cribrosa parameters.

Parameters	Average (SD)	Imprecision	Relative Imprecision
Pore Diameter (µm)	24.2 (1.9)	0.4	1.8%
Pore Diameter SD (µm)	9.8 (0.8)	0.2	2.0%
Pore Aspect Ratio	2.00 (0.11)	0.04	1.8%
Pore Area (µm^2^)	1660 (206)	50	3.0%
Beam Thickness (µm)	48.8 (2.7)	1.0	2.0%
Beam Thickness SD (µm)	16.1 (1.7)	0.7	4.2%
Beam Thickness to Pore Diameter Ratio	2.0 (0.1)	0.1	1.8%

**Table 2:** Relative imprecision of lamina cribrosa parameters in relation to clinical diagnosis.

Parameters	Healthy (n = 8)	Glaucoma Suspect (n = 19)	Glaucoma(n = 12)
Pore Diameter (µm)	2.8%	1.5%	1.2%
Pore Diameter SD (µm)	2.7%	1.6%	2.2%
Pore Aspect Ratio	2.1%	1.7%	1.8%
Pore Area (µm^2^)	3.0%	2.4%	4.2%
Beam Thickness (µm)	2.0%	2.2%	1.8%
Beam Thickness SD (µm)	4.7%	4.7%	2.7%
Beam Thickness to Pore Diameter Ratio	1.9%	1.9%	1.4%

## Discussion

Quantification of the 3D LC microarchitecture in-vivo had been difficult due to a lack of an automated analysis tool. An accurate and reproducible in-vivo analysis of the LC will be useful for examining glaucomatous changes on the microarchitecture of the LC in a large patient population. In this manuscript we demonstrated that an automated 3D LC segmentation analysis tool we recently developed [12] provided highly reproducible information on the 3D LC microarchitecture in a group of healthy and glaucomatous eyes, representing the typical mixture of subjects handled in glaucoma service. The relative imprecision of all parameters was no larger than 4.2%.

The ability to automatically quantify in-vivo human 3D LC had several important advantages and implications. In-vivo imaging does not suffer from distortions due to the loss of pressure (intraocular pressure, intracranial pressure, or blood pressure), distortions in tissue during histology preparation or tissue degradation after death. In addition, in-vivo imaging also permitted repeated scanning and longitudinal analysis, as well as studies comprising of a more representative population than those who donate their eyes. 3D analysis enabled thorough quantification of the complex 3D structure of the LC, which was more comprehensive than 2D or surface-projection studies performed so far in-vivo. The automated segmentation analysis helped remove the subjectivity of manual segmentation and permitted rapid investigation of a large number of eyes.

Conceptually, we expected that parameters generated by 3D analysis would show better reproducibility than those generated by averaging across sequential C-mode slices. Whereas averaging across all sequential C-mode slices does represent the entire visible LC, the measurement could vary due to small shifts in scan angle. For example, shadows due to blood vessels might cause pores to appear in one scan angle, but not another. Therefore, analyzing parameters such as pore count would be not be appropriate in these OCT studies of the LC. As they would only reflect region of visible LC per scan, not some intrinsic property of the LC. Nevertheless, we demonstrated that both 3D and averaging across sequential C-mode provide robust and reproducible measurements of the LC microstructure (Table 1).

Differences in regions of analyzed LC between two different C-mode scans were noted in some of the eyes (Figure 2). The analysis tool was designed conservatively in determining the analyzable LC to insure that the segmented region was indeed part of the LC, and not noise. However, while the LC outlines might be slightly different in consecutive scans, the global 3D microarchitecture of the LC was still preserved (Figure 4).

The pore area and aspect ratio reported in this study were nearly identical to those reported by Ivers et al. from a small cohort of healthy subjects, where adaptive optics (AO−) SLO was used and the pores were segmented manually. [8] Akagi et al., reported similar aspect ratio but larger pore area using manual segmentation of AO-SLO images of both healthy and glaucomatous eyes. [6] This difference might be related to the different disease severity between the studies, analyzable LC, pore selection, and definition of the pore margin. [6] Pore area, as measured in-vivo in our study, was slightly larger than the area reported in histologic studies (∼1460 and 920 µm^2^), which might be related to the tissue shrinking during histological processing, the quantification of the surface pores only or due to the fact that both histologic studies included only healthy eyes. [19], [20] The LC parameters reported in this manuscript are all within 10% of previously published findings using SS-OCT. [12] It is important to note that in the previous manuscript, the segmentation analysis was performed on a single C-mode per eye.

The relative imprecision varied between the diagnostic classes (Table 2). In general, there was a tendency of highest imprecision in the healthy group and lowest in the glaucoma suspects, though for most parameters the range was small. Only the imprecision for pore diameter was significantly higher in healthy compared to glaucoma subjects. The higher imprecision in the healthy subjects may be due to the thicker prelaminar tissue in these subject, which may decrease the scan quality and segmentation at the level of the LC.

The main limitation of this study, similar to most other in-vivo imaging studies analyzing the LC, was related to the ability to capture the entire LC, which was highly dependent on the characteristic of the blood vessels and prelaminar tissue overlying the LC (Figures 1, 2). This inevitable limitation was related to the complex structure of the LC region and the physical properties of the OCT technology. Yet, the low imprecision reported in this study confirmed that the differences in the various parameters between consecutive scans for a given eye was small and therefore microstructural changes could be detected reliably.

In conclusion, automated segmentation for assessing 3D LC microarchitecture demonstrated low imprecision and high reproducibility. This analysis method represented a useful tool for future 3D analysis of the LC in-vivo.
